# Mocetinostat as a novel selective histone deacetylase (HDAC) inhibitor in the promotion of apoptosis in glioblastoma cell line C6 and T98G

**DOI:** 10.21203/rs.3.rs-4170668/v1

**Published:** 2024-04-01

**Authors:** Firas khathayer, Mohammed Mikael

**Affiliations:** University of Mosul; University of Mosul

**Keywords:** Glioblastoma, MGCD0103, Cell line T98 and C6, Apoptosis, differentiation, Invasion and Metastasis

## Abstract

Histon deacetylase (HDAC) enzyme is one of the enzymes involved in regulating gene expression and epigenetic alternation of cells by removing acetyl groups from lysine residue on a histone, allowing the histones to wrap the DNA more tightly and suppressing a tumor-suppressing gene. HDAC inhibitors play an important role in inhibiting the proliferation of tumor cells by restricting the mechanism of action of HDAC enzyme, leading to the addition of acetyl groups to lysine. Mocetinostat, also known by its chemical name (MGCD0103), is a novel isotype selective HDAC enzyme that explicitly targets HDAC isoforms inhibiting Class1(HDAC 1,2,3,8) and Class IV (HDAC11) enzymes. It was approved for treating the phase II trial of Hodgkin’s lymphoma in 2010. Our study revealed that different doses of Mocetinostat inhibit the growth of glioblastoma cells, metastasis, and angiogenesis and induce the apoptosis and differentiation of glioblastoma cells C6 and T98G. Western blot has shown that MGCD0103 has many biological activities to control glioblastoma cancer cells. MGCD0103 can modulate the molecular mechanism for several pathways in cells, such as inhibition of the PI3K/AKT pathway and suppression of HDAC1 enzyme activity in charge of many biological processes in the initiation and progression of cancer. The high doses of Mocetinostat drug significantly induce apoptosis and suppress cancer cell proliferation through increased pro-apoptotic proteins (BAX) and a down level of anti-apoptotic proteins(Bid, Bcl2). Also, the mocetinostat upregulated the expression of the tumor suppressor gene and downregulated the gene expression of the E2f1 transcription factor. Additionally, MGCDO103-induced differentiation was facilitated by activating the differentiation marker GFAP and preventing the undifferentiation marker from expression (Id2, N-Myc). The MGCD0103 is a potent anticancer drug crucial in treating glioblastoma cells.

## Introduction

Genetic alternation is not only involved in cancer cells, but the epigenetic modifications that cause gene expression alternation without changing the DNA sequence are responsible for carcinogenesis for many cancer cells. Recent studies indicate that epigenetic modification (DNA methylation, Histone modification, and Non-coding RNA deregulation) is the cause of initiation and cancer development (Li et al.,2010; Sharma et al.,2010). Histone modification has an essential role in regulating gene expression; it causes upregulation of oncogenes and DNA repair genes by histone acetylase and deacetylase (Perri et al.,2017). Histone deacetylase (HDAC) is a family of enzymes considered essential in the epigenetic regulation of gene expression that leads to the control of diverse cellular activities, such as growth cells, apoptosis, invasion cells, and angiogenesis. These enzymes usually remove the acylal group from the histone tail, leading to silent transcriptional gene of tumor suppressor gene (Minucci &Pelicci,2006). HDAC in humans is classified into four classes depending on how closely their sequence resembles those of yeast HDACs. Class I, II, and IV HDAC have Zn2 + dependent enzymes, while class III comprises Zn^2+^ independent (Lucio-Eterovic et al.,2008). HADC1 belongs to HDAC class I, increasing their expression in many cancer cells. Also, the study found that express this enzyme elevated in glioma cells during tumor progression, and downregulation of HDAC1 suppressed cell proliferation and invasion of glioblastoma cell line U251, T98g cells (Wang et al.,2017). Epigenetic therapy controls the regulation of epigenetic alternations that occur in tumors and convert to the normal epigenome. Some epigenetic inhibitors, including the HADC inhibitor, have been approved by the U.S. Food and Drug Administration (FDA) (Chen et al.,2013).

HDAC inhibitors have been shown to influence many pathways to inhibit growth cells and induce apoptosis and differentiation. Still, HDAC inhibitors maintain poor performance in cancer treatment because these inhibitors are almost nonselective HADC inhibitors and suppress multiple classes of HADCs, which result in toxicity and limit therapeutic efficacy (Zhang et al.,2016). One of the new classes I selective HDAC inhibitors, mocetinostat, also known as MGCD0103, specifically targets the suppression of human class I HDAC. (Fournel et al.,2008). MGCD0103 is a benzamide histone deacetylase inhibitor undergoing preclinical trial for the treatment of many cancer cells in vitro and in vivo, such as the colon (Sikandar et al.,2010), prostate (Zhang et al.,2016), Human epithelial cancer cell (Fournel et al.,2008), and Hodgkin’s lymphoma (Younes et al.,2011). Also, MGCD0103 has a high ability to inhibit HADC2, HADC3, and HADC11 in vitro and increase acetylation on histone tail, which induces apoptosis and inhibits growth cells for a variety of cancer cells (Fournel et al.,2008).

Glioblastoma is the most dangerous malignant primary brain tumor that affects the adult’s central nervous system. They arise from glial cells and represent grade IV glioma (Louis et al.,2016). GBM remains one of the deadliest tumors, and its treatment methods remain limited (Stupp et al.,2005). Histone deacetylase inhibitors have a great interest in cancer treatment because they can alter the outcome of RNA sequences to induce apoptosis. Characteristics of Glioblastoma, representing increased proliferation, angiogenesis, invasion, and migration, triggered apoptosis and differentiation, are usually targeted via HDAC inhibitors. HDAC inhibitors are a promising class of therapeutic drugs being researched for treating several tumors, including GBM (Lee et al.,2017).

In the present study, we investigated the role of Mocetinostat in the treatment of human glioblastoma cell lines, and we will show inhibitory activity for Mocetinostat in suppressing cell proliferation, migration, inducing apoptosis, and differentiation of glioblastoma in two cell lines T98G and C6 in vitro. MGC0103 might be a promising anticancer drug in the treatment of glioblastoma.

## Materials and Methods

### 1.2 Cell culture and treatment

Human glioblastoma cell lines T98G and rat glioma C6 cells were obtained from American-type culture collection (ATCC, Manassas, VA, USA). Both cells were grown in RPMI 1640 containing 1% penicillin-streptomycin and 10% fetal bovine serum (GIBCO/BRL, NY, USA). Cultures were maintained in a humidified atmosphere with 5% CO2 at 37°C. Cells were treated with different concentrations (0.0, 0.5, 1.0, 1.5, 2.0, 2.5) μM of HDAC inhibitor (MGCD0103) that was purchased from Selleckchem.com (Selleck Chemicals, Houston, TX, USA). The drug was dissolved in dimethyl sulfoxide (DMSO) to get the final concentration (5.0mM) like a sock solution. The stock solution was stored at −20°C.

### 2.2 A 3-(4,5-dimethylthiazol-2-yl)-2,5-diphenyltetrazolium bromide (MTT) cell proliferation assay

The MTT assay is the best method to measure the viability of cells and the cytotoxic effects of drugs. Cellular metabolic activity of C6 and T98G was measured depending on the manufacturer’s instructions (ATCC, Manassas, VA, USA). Briefly, 0.5 × 10^4^ cells/well were seeded into 96-well plates containing growth medium RPM1 with supplements (10% PBS and 1% penicillin and streptomycin) and incubated with 5% CO2 at 37°C. After the growth cells got 70% confluence, the current medium was replaced with a new medium containing varied concentrations (0, 0.5, 1.0, 1.5, 2.0, 2.5) μM of MODC0103 for 48 h. Then, the 10μL MTT solution (ATCC, VA, USA) was put in each well and grown for 4h. Detergent reagent (ATCC, Manassas, AV, USA) was applied to each well in quantities of 100 mL to dissolve MTT formazan crystals., a wavelength absorbance was measured using a microplate reader (Bio-Rad, Hercules, CA, USA) at 570 nm, absorbance was recorded, and at 690 nm absorbance of the background was subtracted. All experiments were in triplicate.

### 3.2 In vitro differentiation cancer cells assay

The different concentrations of MGCD0103 were used to identify the ability of mocetinostat to induce astrocytic differentiation in glioblastoma cell lines C6 and T98G. Using 6 well plates, cells were cultivated in growth medium RPMI-1406 with 10% FBS and 1% Penicillin/Streptomycin. The cultures were placed in the incubation with 5.0% CO_2_ and 37 C for 24h. After the culture medium was removed, the cells cultured in the medium were supplemented with different concentrations of MGCD0103. The cells were placed in the incubation conditions of 5% CO_2_ at 37 C for 48h. After treating the cells with drugs, the culture medium was aspirated and washed twice with BPS. After that, the cells were fixed and stained using a Kwik-Diff^™^ kit according to the protocol of the manufacturer (Thermo-Scientific, MO, USA). The samples were examined under light microscopy to watch the differentiation features of cells.

### 4.2 Detection of Morphological characteristics using Apoptosis assay

The morphological feature of apoptosis cells was achieved by right staining. Both cell lines C6 and T98G at density 0.5×104 were cultured in 6 wells containing RPMI-1460 supplement with 10% FBS and 1% Penicillin /Streptomycin, then incubated in the condition of 5% CO2 at 37 C for 48 h. After removing the old medium, 2mL of the new medium supplement with Each well received different doses of MGCD0103 before being incubated for 48 hours. The supernatant was discarded and washed twice with PBS. Then, the cells were fixed and stained utilizing a Kwik-Diff^™^ staining kit following instructions provided by the manufacturer (Thermo-Scientific, St. Louis, MO, USA). The morphological features of apoptosis cells were imaged under a light microscope (Olympus, Pittsburgh, PA, USA). Image J was used to measure the percentage of apoptotic cells. The apoptosis rate was measured after counting 300 cells from each treatment.

### 5.2 Identification of biochemical apoptotic populations using flow cytometry and Annexin FITC/PI double staining.

a Fluorescein Isothiocyanate (FITC)-Annexin V and propidium iodide (PI) staining Apoptosis were examined to detect the Biochemical characteristics of apoptotic cells using Apoptosis Detection kit (BD Biosciences) according to the manufacturer’s instructions. Both cell lines (C6 and T98g) were cultivated independently in 6 wells and subjected to various concentrations of MGCD0103 (0.0, 0.5, 1.0, 1.5, 2.0, 2.5) μM for 48 h. Once the cells were harvested and centrifuged at 2500x for 5 min, they were washed cold BPS twice and resuspended in 100μl 1x binding buffer (0.1 M HEPES, NaOH, PH 7.4,1.4 NaCl, and 25Mm Cacl2). The cells were transferred to a 5mL polystyrene culture tube and stained with 5μl of Annexin-V-FITC and 5μL of PI. All tubes were incubated for 15 min at darkroom temperature for the staining of cells. Then, 400μL of binding buffer was added to each tube and analyzed by flow cytometry using an Epics XL-MCL Flow Cytometry (Beckman Coulter, Fullerton, CA, USA). Three duplicates of each experiment were carried out. The cell populations were identified on the plot as a red dot, with viable cells (FITC−/PI−) appearing in the left lower quadrant (Q3), early apoptotic cells (FITC+/PI−) were displayed in the right lower quadrant (Q4), and necrotic and late apoptotic cells (FITC+/PI+) were presented in the right upper quadrant (Q2). The necrotic cells were seen in the left upper quadrant (Q1).

### 6.2 Scratch wounding migration assay

Determining the ability of MGCD0103 to suppress the motility of cells was evaluated by migration assay. Both 2×10^5^ T98G and C6 cells were grown in 6 well after the cells approximately reached a confluence rate of 90%. A 200-L sterile plastic pipette tip was used to gently scrape the cell monolayers in the center of the well. To eliminate the floating and debris cells, the gap was twice rinsed with a growing media. The old medium was removed from each well and a fresh medium containing various concentrations of MGCD0103 was added to each well and incubated with 5% CO_2_ at 37 C for 24 h. Then the medium was discarded and fixed with 70% alcohol. Inverted light microscopy with a camera (BioTeck Instruments, Inc, VT, USA) was used to examine the cells’ movement into the wound area. and the distance between the scratches was analyzed by Image J. To calculate the percentage of wound closure, the formula below was used: Closure of a wound is equal to [1-(wound area at Tt/wound area at T0) 100. The wound’s creation time is T0, and the time since it was caused is Tt. Three times the tests were run.

### 7.2 Matrigel Invasion assay

Briefly, C6 and T98G cells were cultivated in a 24-transwell chamber supplied with 8.0μm pore size polycarbonate filters (Corning Life Science, Corning, NY, USA) covered in 200 μL Matrigel (Corning Life Science, Corning, NY, USA) at a final concentration 1.0 mg/mL, which was allowed to solidify at 37 C for 4h. 1×10^5^ cells were suspended with 100 μL of growth medium-free serum and placed into the upper chamber of the transwell. In the lower chamber of the transwell plate, 600 μL of RPMI-1640 growth medium containing 10% FBS and 1% penicillin + streptomycin supplement with various concentrations of MGCD0103 was added and then incubated for 2 days in 5% CO2 at 37 C for 48h. At the end of incubation, the cells had invaded the Matrigel and attached to the lower surface of the filter.

Consequently, the medium from the upper chamber was discarded, and the cell migration was fixed by alcohol 70% for 1 min. Once the alcohol was removed, the cells were stained with 0.1% crystal violate for 15 min. Then, the cells were washed with double distilled water. By swabbing cotton, the non-migration cells were eliminated. Light microscopy was used to examine the cells’ passage through the Matrigel and onto the lower surface of the filter. (Leica Microsystems, Inc, USA) and the percentage of cells’ migration was quantified using the Image J program (National Institutes of Health, Bethesda, MD, USA).

### 8.2 Invitro network angiogenesis assay

To study the effect of various concentrations of MGCD0103 on the inhibition of angiogenesis, we achieved network angiogenesis in vitro by using the Co-Culture Tube Formation Assay kit (Ibidi, Gräfelfing, Germany) as described in the company’s instructions. The Matrigel (10 μL) was placed in a 15-well chamber slide and incubated with 5% CO_2_ at 37 C to solidify for 1h. Each of the C6 and T98G cells at a density of 2.5 ×10^4^ was separately mixed with 10^4^ human mammary epithelial (HME) cells as co-culture, then they were added to each tube containing 50 μL RPMI and incubated for 6 h to network formation. After the culture medium was aspirated off the Matrigel surface, 50 μL of growth medium supplement of different concentrations was added to each well-containing network. The chamber slide was incubated for 24 h in 5% CO_2_ and 37C. After that, the culture medium was expelled. Cells were fixed with methanol and the image was observed by light microscopy (Leica Microsystems, Inc, USA). The tube information was counted by Image J software (National Institutes of Health, Bethesda, MD, USA).

### 9.2 Proteins extraction by SDS-PAGE assay

Protein extracted from both cell lines C6 and T98G were cultured in (60mm) of growth medium RPIM with 10% FBS, 1% Penicillin /Streptomycin, and incubated in 5% CO_2_ at 37 C. After the cells cultured reached confluence, the culture medium was removed and replaced with a culture medium supplement with various concentrations of MGCD0103 for 48 h at 37 C. Once the treatment period was finished, the pellet from the cells was scraped and washed twice with ice-cold PBS. The cells were centrifuged at 2500rpm for 5 min and discarded in their supernatant. After that, the pellet cells were lysate with 250μL of cold RIPA buffer (25mM Tris•HCl pH 7.6, 150mM NaCl, 1% NP-40, 1% sodium deoxycholate, 0.1% SDS) (Thermofisher, IL, USA) and kept on ice for 30 minutes. The test tubes were shaken five times by vortex. After being lysate, they were centrifuged at 13,000rpm for 15 min at 4 C. The supernatant-containing proteins were then transferred to tubes and kept at −20 C for further analysis.

### 10.2 Western blot

The protein concertation was determined with BCA and Coomassie blue. The equal amounts of protein from each sample mixed with loading buffer and mixtures were boiled at 100 C for 5 min. The samples were separated by electrophoresis on 4–20% of SDS-PAGE gel for 1.5 h. After electrophoresis, the blot was transferred from the gel into the PVDF membrane (Thermofisher, IL, USA). Block with 5% nonfat dry milk in PBS-Tween 20 (0.05%, v/v) for 1 hour at room temperature. Following the membrane were incubated with the primary antibody overnight. The next day, the membrane was washed five times with PBS. Subsequently, a Suitable horseradish peroxidase-conjugated secondary antibody was incubated with the membranes. that was added to the membrane for 1h at room temperature. After washing with (PBST) five times at room temperature, the membrane was incubated with enhanced electro chemiluminescent for 5 min the results were acquired by X-ray film.

### 11.2 Antibody

The antibody anti-B-actin (#4967) (Cell Singling, USA) was purchased from Cell Signaling (Cell Signaling, MA, USA). The other primary antibodies used in this study were Bax(sc-4780), Bcl2(sc-7382), Bid(sc-6539) HDAC1, (sc-7872), Caspase 3(sc-7148), MMP9 (sc-13520), MMP 2(sc-1359), GAFP(sc-6170), pRB(sc-12901(), Rb(sc-15)N-Myc(sc-42), Id2 (sc-489), VEGF (sc-7269), EGFR2 (sc-271847), P-BAD(sc-133356), pAKT-1(sc-514032) were purchased from Santacruz Biotechnology (Santa Cruz, CA, USA) PI3K (GTX50858) was purchase from Gene Tex, USA, AKT(6744) was purchased Bio vision, USA). Horseradish peroxidase-conjugated secondary anti-rabbit was purchased from Santa Cruz Biotechnology (Santa Cruz, CA), and anti-mouse (Santa Cruz, CA, USA)

### 12.2 Statically analysis

Data were represented as the mean standard error (± SEM). All experiments were repeated three times. In all experiments, results were analyzed using GraphPad Prism software (one-way ANOVA). Statically differences between groups were considered relevant at P > 0.05. All measurements were compared with the control. P*<0.05, P**<0.01,p#<0.001

## Results

### 1–3 Evaluation effect MGCD0103 on cell viability by MTTS assay

**Figure 1:**
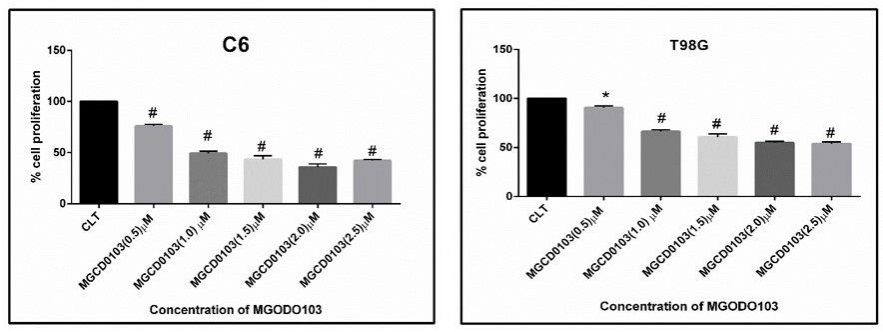
MTT assay showing the effect different concentrations of MGCD0103 on cell proliferation of C6 and T98G glioblastoma cells line by MTT assay. **A)** Diagram significantly demonstrate decrease in the cell viability of C6 glioblastoma cell line after 48h treatment with different concentrations of MGCD0103. **B)** Diagram significantly demonstrate decrease in the cell viability of T98G glioblastoma cell line after 48h treatment with different concentrations of MGCD0103.

To investigate the ability of the anti-tumor activity of MGCD0103 on cell proliferation of glioblastoma cell lines C6 and T98G, we examined cell viability for both cell lines by a 3-(4,5-Dimethylthiazol-2-yl)-2,5-diphenyltetrazolium bromide) MMT assay (Fig. 1). The glioblastoma was treated with various concentrations of MGCD0103 (0.5,1.5,2.0,2.5) μM for 48h. The MTT results showed that the viability of cells in both cell lines was significantly decreased with the increased dose of MGCD0103 compared with the control. The percentage of inhibition in growth cells was different based on the dose-dependent manner of drugs. Low concentrations of MODC0103 (0.5 μm) did not show significant effects on the growth of glioblastoma cell lines C6 and T98G, while other concentrations of MGCD0103 (1.0,1.5,2.0,25 μm) significantly reduced ((P < 0.005) growth cells in both cell lines. The higher inhibitory ratio in the proliferation of cells was with doses (2.0, 2.5) μM, which reached 35% and 41.6%, respectively in C6, while the inhibitory ratio was 54% and 53%, respectively in T98G.

### 2.3 Differentiation assay

**Figure 2:**
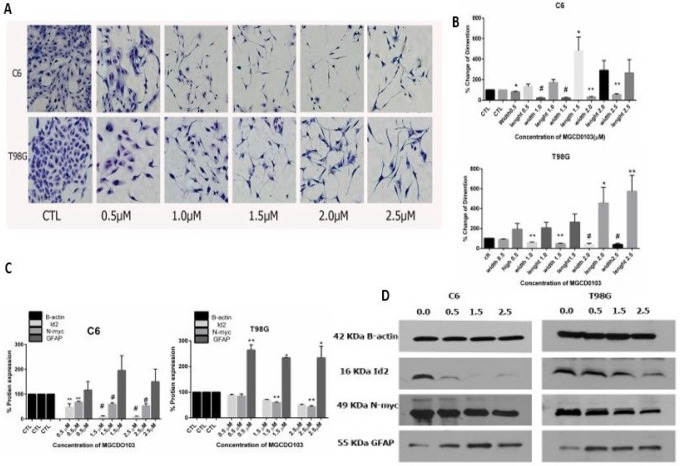
Effect different concentrations of MGCD0103 on morphological and biochemical features of differentiation of glioblastoma cells line. **A)** In situ wright staining show morphological features of differentiation C6 and T98G glioblastoma cells line after treatment with different concentrations of MGCD0103 for 48h. **B)** Histogram is shown measurement of morphological features of differentiation of glioblastoma (cell width, cell length). Three experiments are compared with control P*<0.05, P**<0.01.P# <0.001. **C)** Detect of expression of proteins involved in differentiation by western blot **D)**. Bar show determent percentage of proteins mediated in differentiation after treatments P*<0.05, P**<0.01, P# <0.001.

The MGCD0103 potently affects the differentiation of glioblastoma cells C6 and T98G. The morphological features of differentiation cells were small and retracted cell bodies with thin, elongated, and branched processes. The changes in morphological characteristics appeared after the cells were treated with a variety of doses of MGCD0103 for 48h compared with an untreated cell (control) that kept the vast cell body with short processes (Fig. 2). The concentrations of MGCD0103(1.5) μM significantly contributed to an increase of cell length and decrease of cell width in C6. In contrast, the perfect concentration in increased length and width of cells was (2.5) μМ in T98G. The length of cells reached 481% and the width of cells 21% in C6, while the length of cells got 575%, and the width of cells was 38% in T98G. The protein expression in differentiation was achieved by the western blot after treatment. The biomarker of astrocytic differentiation in both cell lines (GFAP) was upregulated, while the proteins mediated in the dedifferentiation of cells (Id2, n-MYC) were downregulated after treatment with various diosgenin concentrations.

### 3.3 Wright staining

**Figure 3:**
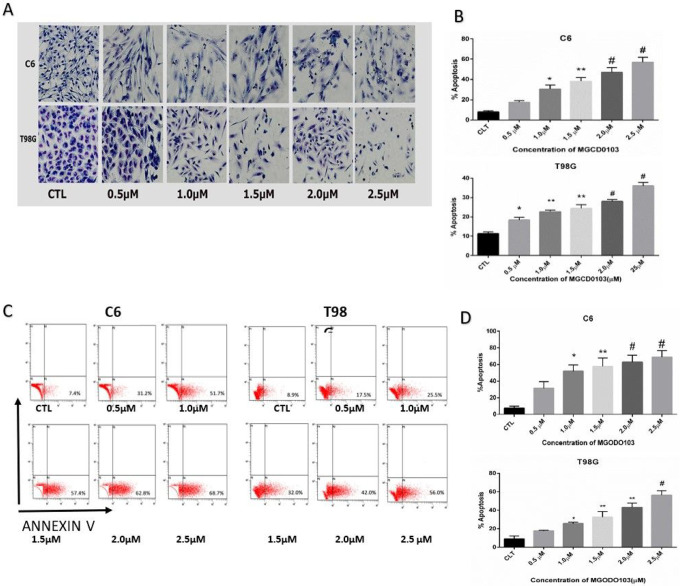
Effect different concentrations of MGCD0103 on the apoptosis of glioblastoma cells line **A)** *In situ* Wright staining to examine morphological features of apoptotic cells and demonstrate increase in percentage of apoptotic cells after treatment with different concentrations of MGCD0103 on growth of C6 and T98G for 48h. **B)** The percentage of apoptotic cells represent in bar diagrams based on in situ wright staining. Three independent experiments compared with control P*<0.05, P**<0.01, P#<0.001. **C)**Biochemical features of apoptotic cells were measured by Annexin V-FITC/PI staining assay and demonstrate effect different concentrations of MGCD0103 on growth of C6 and T98G after treatment for 48h **D)** Quantitative apoptotic cells showed in bar diagram base on annexin v positive stating in A4 quadrant. Three experiments compared with control P*<0.05, P**<0.01.P#<0.001.

The morphological signs of apoptotic cell death for both cell lines C6 and T98G were detected by in *situ* wright staining assay (Fig. 3). The apoptosis percentage increased after treatment with different concentrations of MGDC0103. The morphological features for apoptosis have represented blebbing, shrinkage of cell volume, condensation of chromatin and fragmentation, nuclear fragmentation, and membrane-attached apoptotic bodies. These features significantly increased(p < 0.005) in apoptosis with the high doses of MGDC0103 (20, 25) μm. The percentage of apoptotic cells that led to apoptosis was 45% and 58% in doses (20, 25) μM, respectively, in C6 cells. In contrast, the apoptosis ratio reached 28%.36% in concentrations (20, 25) μM respectively in T98G compared with control cells. The results demonstrated that MGCD0103 plays a vital role in the induction of apoptosis.

### 4.3 Flow cytometry

The biochemical characteristics of apoptosis in both cell lines C6 and T98G were detected via annexin V-FIC/PI staining and analyzed by flow cytometry (Fig. 3). The early apoptotic population of Annexin V positive cells in A4 has increased after treatment with different concentrations of MGCD0103. This increase depended on the percentage of concentration added to cells. The data indicated that the inhibition rate significantly increased in high concentrations (20, 25) μM, which reached 62.6% and 68.75%, respectively, in C6, and induced apoptotic increase with increased doses. In comparison, the percentage of apoptotic cells was 43% and 56.3% in 20.25 μM, respectively in T98G.

### 5.3 Role protein involved in inducing activated extrinsic and intrinsic apoptosis pathway apoptosis in glioblastoma.

**Figure 4:**
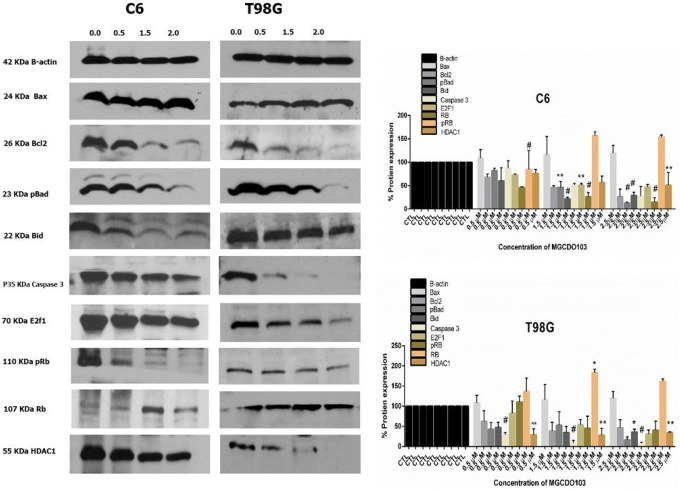
Western blot for measurement expression of the proteins involved in cell proliferation and apoptosis after treatment with different concentrations of MGCD0103 in C6 and T98G glioblastoma cell line **A)** Western blot show expression of proteins involved in apoptosis in both cell line ce6 and t98g. **B)**Western blot analysis for amount of proteins involved in apoptosis and cell proliferation for both cell line glioblastoma the results showed in two independent experiment and mean± SE P*<0.05,P**<0.01.P# <0.001

The morphological and biochemical features of apoptosis in glioblastoma cells after treatment with different concentrations of MGCD0103 have induced the mitochondria to activate the extrinsic and intrinsic pathways. These pathways are involved in many proteins’ expressions. Western blot assay was used to evaluate the expression of these proteins and the expression of B-actin that was used as a control (Fig. 4). The results of the western blot found that MGDC0103 activated extrinsic pathways by increasing the levels of Caspase 8 and t-bid. Also, the varied concentrations of MGCD0103 highly contributed to inducing intrinsic pathways by upregulating Bax, which represents a pro-apoptotic protein, and downregulating bcl2, which is anti-apoptotic. The increase in Bax Bcl2 usually contributes to the induction expression of caspase 3 and AIF. To demonstrate whether MGCD0103 contributes to an increased level of caspase 3. The results found that the expression of procaspase 3 has decreased in both cell lines C6 and T98G after treatment with the drug, while cleavage to caspase 3 has increased after treatment. Also, we investigated the influence of varied doses of MGCD0103 in induction cell cycle arrest in both cell lines by changing protein expression mediated in cell cycle arrest. Retinoblastoma’s suppressor gene (Rb) which represents a marker of cell cycle arrest was upregulated after treatment with different concentrations of MGCD0103 in a dose-dependent manner. In contrast, the expression of pRB and E2F involved in the S phase in cell cycle progression was low after treatment. HDAC1 enzyme is essential in epigenetic alternation, which requires cell proliferation and inhibits apoptosis. By western blot, we investigated the effect of MGCD0103 on HDAC1 protein expression in both cell lines. The level of HDAC1 protein was significantly reduced in two glioblastoma cell lines C6 and T98G after dealing with various concentrations of MGCD0103.

### 6.3 Wound healing

**Figure 5:**
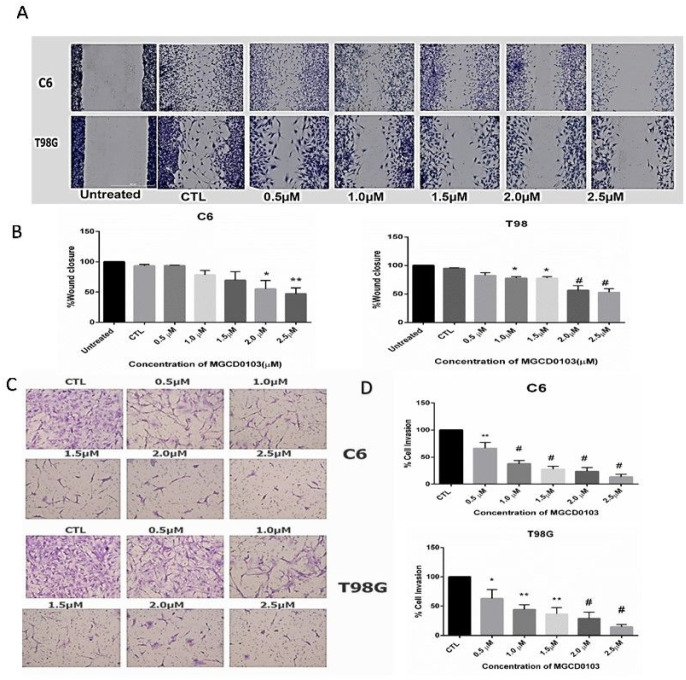
Inhibitory effects of MGCD0103 on cell migration and invasion of T98G and C6 glioblastoma cells line **A)** Wounding healing assay to measure effect different concentrations of MGCD0103 on cell migration of glioblastoma cell lines and show decrease in percentage of cell migration after treatment for 24h **B)** Ratio the migration for C6 and T98G glioblastoma cell after treated with MGCD0103. All the experiments are compared with control P*<0.05, P*<0.01 **C)** Transwell chamber assay to evaluate the different concentrations of MGCD0103 on cell invasion that demonstrate reduction in cells invasion for 48h from treatment **D)** rate the of cell invasion represent in bar after treatment of different concentration of MGCD0103. All three experiments are compared with control P*<0.05, P*<0.01P#<0.001.

The effect of anti-cancer drugs MGCD0103 on cell migration was examined. The result of the wound healing assay showed that various concentrations of MGCD0103 had a restrained influence on the motility of both glioblastoma cell lines compared with the control that increased cell migration (Fig. 5). The high concentration of MGCD013 highly inhibited cell migration compared with a low concentration of MGCD0103. The wound closure rate had reduced to 55% and 47% when the C6 was treated with (20, 25) μM, respectively. A similar reduction in migration ability was noticed when T98G cells were treated with different concentrations of MGCD0103. The number of cell motility had decreased to 56% and 52% respectively, in concentrations (20, 25) μM of MGCD0103.

### 7.3 MGCD0103 inhibits the invasion cells

We investigated whether the MGCD0103 could inhibit glioblastoma cell invasion across the extracellular matrix and performed a transwell invasion assay. The results of the invasion assay showed that MGCD0103 suppressed cell invasion in both cell lines C6 and T98G compared with control in a dose-dependent manner (Fig. 5). The number of cell invasions through transwell-coated Matrigel had reduced with increasing MGCD0103 concentrations in cell lines C6 and T98G. The inhibitory percentage of cell invasion in C6 reached 20.3% and 13.3% with (20, 25) μM, while the T98G cell invasion rate was 28% and 14% with 20% and 25%, respectively, compared with untreated cell control. It has been widely recognized that the migration and invasion of glioblastoma cells always mediate the activation of the PI3k /AKT pathway. We performed a western blot to explore the role of MGCD0103 on the expression of PI3k and phosphorylation of Akt. The western blot results have shown that the expressions of two proteins expression were downregulated after treatment with MGCD0103 compared with untreated cells. Also, the expression of MMP2 and MMP9 is required for invasion by the degradation of the extracellular matrix. We assessed the effect of MGCD0103 on the expression of these two proteins by western blot. The results demonstrated that MGCD0103 could inhibit the breakdown of the extracellular matrix by downregulating protein levels of MMP2 and MMP9. The expression of MMP2 and MMP9 was significantly reduced after treatment with 2.5 μM of MGCD0103 in both cell lines c6 and T98G. These results indicate that MGCD0103 might be essential in inhibiting invasion and migration of glioblastoma cell lines.

### 8.3 Angiogenesis assay

**Figure 6:**
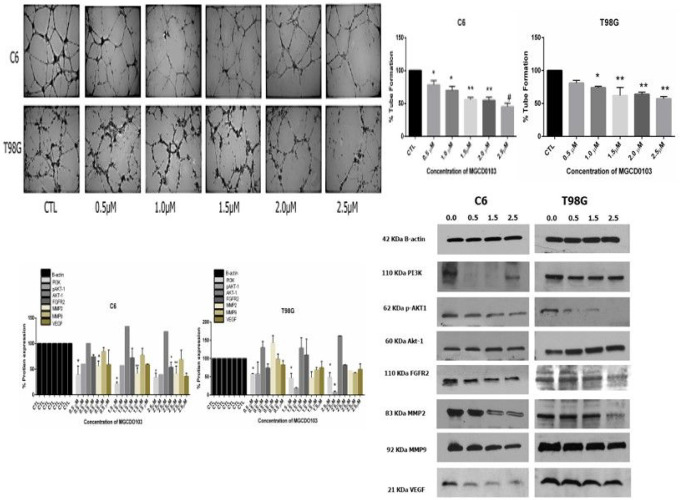
In vitro coculture angiogenesis assay to measure effect different concentrations of MGCD0103 on tube formation growth **A)** Tube formation test demonstrated significant decrease in number tube formation after treatment co culture HME with C6and T98G respectively with different concentrations of MGCD0103 for 48h **B)** the percentage of tube formation subjected in bar histogram r. All three experiments compared with control P*<0.05, P**<0.01.P#<0.001) **C)** Western blotting showed changed in gene expression for proteins involved in invasion, migration and angiogenesis in C and t98g glioblastoma **D)** The quantitative relative expression of proteins involved in migration, invasion, and angiogenesis represent n histogram after treatment with MGCD0103.

To examine the inhibitory effects of MGCD0103 on in vitro angiogenesis assay, cell lines C6 and T98G were treated with various concentrations of MGCD0103. The MGCD0103 had different cytotoxicity effects depending on doses of MGCD0103 and the type of glioblastoma cells. The drug has a critical role in decreasing the number of tube formations of T98G and C6 glioblastoma cell lines compared with the control (Fig. 6). Also, the high doses significantly reduced the number of tube formations compared with low concentrations and control cells. The percentage of tube formations represented in bar graphs clearly showed that coculturing HME with C6 decreased to 54% and 45% with (20, 25) μM, respectively, while the coculturing HME with T98G was 57%, 64% with (20, 25) μM. VEGF plays a vital role in angiogenesis. EGFR attaches to the cell surface receptor and activates angiogenesis. We studied the effect of various concentrations of MGCD0103 on the activation of VEGF and EGFR expression. The results have shown the MGC0103 significantly downregulated the expression of these two proteins. Since rapamycin (mTOR) and NFkB are proteins mainly involved in the induction of VEGF mediated in angiogenesis by activating PI3K/AKT phosphorylation.

## Discussion

MGCD0103, one of the HDAC inhibitors, has a broad spectrum as antitumor activities against different types of cancer cells *in vivo* and *in vitro* without any cytotoxicity on animals (Founel et al., 2014). Histone deacetylase-1 (HDAC 1) is overexpressed in human glioblastoma, and they participate in varied biological processes including cell growth, invasion, and migration of cells. Knockdown of HDAC1 can inhibit the initiation and progression of cancer cells(Was et al., 2019;Wang et al.,2017). The MGCD0103 is an isotype selective HDAC inhibitor and targets HDAC1,2,3,11(Zhou et al., 2008). The present study demonstrated that MGCD0103 has an influential inhibitory role in treating two glioblastoma cell lines C6 and T98G by inducing apoptosis, differentiation, and inhibiting angiogenesis. The study has also shown a high ability of MGCD0103 in the prevention of the spread of cancer by reducing invasion and migration cells through the capability of MGCD0103 in the suppression activity of HDAC1. In addition, this study conducted the knowledge molecular mechanism of MGCD0103 in the induction of apoptosis, differentiation, and inhibition of migration and invasion cells in a dose-dependent manner.

Apoptosis is a biological process aim to eliminate proliferation cells. The western blot results revealed that Mocetinostat played a role in induction apoptosis in glioblastoma through the suppression of HDAC1 involved in regulating different proliferative pathways. Caspases induce apoptosis via cleaving specific sets of substrates, and apoptotic pathway of mitochondria is the main pathway for stimulating cell death in cancer cells through activating caspase (Liao et al.,2020). Mocetinostat were promoted the intrinsic and extrinsic pathway by increasing proapoptotic proteins (Bax) and decreasing protein expression (Bcl2 and p-Bad and bid) inducing the proliferation of cancer cells. The increase in protein expression of Bax/Bcl2 led to suppress of cells’ viability through releasing cytochrome C. As it’s known cytochrome C is considered an essential agent in the activation of caspases 3 that leads to the cleavage PARP-1 and ICAD involved in apoptosis cells (McIlroy et al.,1999: Affar et al., 2001). Also, HDACi has a significant role in regulating the activity of the Rb/E2f1 pathway, and it has vital role in initiating DNA replication and cell cycle activity (Joseph et al., 2001). Hyperphosphorylated pRB causes an increase in cell proliferation in many cancer cells through the increase of expression E2F1 and cyclin D1 activity required in cell cycle progression (Zhao et al.,2005), while hypophosphorylated RB associated with E2F1 leads to suppression of gene transcriptions responsible of cell cycle progression (Attwooll et al.,2004). HDACi largely suppresses Hyperphosphorylated pRB activity by inhibiting the expression of cyclin D and causing an increase in the hypo-phosphorylation of RB (Kenta et al.,2018). Our study appeared that the Mocetinost could also induce apoptosis by suppressing the oncogenic E2F1 transcription factor and increasing the expression of RB protein. The previous study found that Mocetinostat could induce apoptosis in liver cancer by downregulating the antiapoptotic protein E2F6 and upregulating the expression Bad in two prostate cell lines(DU-145, PC-3) in vivo and in vitro (Zhang et al.,2016). On the other hand, HDAC1 has an influential role in the promotion of the migration, invasion, and angiogenesis of glioblastoma cells through activation of the PI3K/Akt signaling pathway in vivo and in vitro, so the knockdown of HDAC1 might lead to the suppression of the expression of p-Akt proteins (Li et al.,2018). Also, the previous study found that the suppression of the HDAC1 expression led to the upregulation of the level of cleaved Caspase 3, BAX, and E-Cadherin, and the downregulation expression of MMP9, SNAIL, and TWIST1 in T98G and U251 glioblastoma cell lines (Wang et al., 2017). Our results of the western blot demonstrated that HDACi was significantly suppressed expression of HDAC1 when glioblastoma cells were treated with MGCD0103. The mechanism of MGCD0103 for inhibition of HDAC1 occurs through the attachment of the free amino group of the benzamide of MGCD0103 with the zinc ion in the active site of the HDAC enzyme. This attachment cause inhibition of enzyme activity and induce apoptosis (Fournel et al.,2008. MGCD0103 exerts anti-cancer activity by suppressing the growth of liver cancer cells in vitro by downregulating the expression of Bcl2 and the upregulation of proapoptotic proteins Bax and Bim. At the same time, another study observed that the role of MGC0103 in the inhibition of the proliferation of cancer cells occurs by supporting HDAC1, which is involved in the increase of proliferation of tumor cells. (Liao et al.,2020).

On the other hand, the present study also found that inhibiting HDAC causes the activation of the transcription factors for genes responsible for cell differentiation (Ecker et al.., 2013). The Glial brillary acid protein (GFAP) represents a marker of differentiation cells in glioblastoma, and it is presented in low levels in glioblastoma cells (Lee et al., 2005). The HDAC enzyme suppresses GFAP expression in glioblastoma cells (Morita et al., 2009). The histone deacetylase inhibitors (HDACi) promote differentiation in the human glioblastoma cell line via the decrease of HDAC level and the increase of histone acetylation induced GFAP expression in primary human astrocytoma (Svechnikova et al., 2008; Kanski et al., 2014;). HDAC also increases n-Myc transcription factor expression that in turn overactivation Id2 oncogene in cancer cells (Liu et al.,2017; Yoshikawa et al.,2007). Id2 significantly play an important role in inhibition differenation and promotion growth cell in glioma cell (Yun etal.,2005). Our data found that Mocetnostate largely contributed to the upregulation of GFAP protein and downregulation of Id2, n-Myc. Khathayer et al., 2020 showed that a combination of SAHA and 4HPR increased GFAP and deceased Id2, c-Myc in the glioblastoma cell line C6 and T98G by downregulating of HDAC. Other studies indicated that other HDACi such as 4-PBA, TSA, and VPA promoted the differentiation in glioblastoma cell lines by increasing the expression of GFAP proteins that led to enhancing gap-junction communication between tumor cells and decreased expression of Id2 (Svechnikova et al, 2008; Asklund et al., 2004; Roy et al.,2011).

Cell migration is a set of multicomplex steps that start in response to migratory and chemotactic stimuli by forming protrusion in the cell membrane called invadopodia protostomes(Yamaguchi and Condeelis,2007). In the current study, Mocetinostat has been shown to have high activity in the inhibition of invasion and migration of glioblastoma cells by suppressing PI3K/AKT signaling pathway that is considered one of the most critical intracellular signaling pathways involved in cancer progressions such as invasion, migration, and angiogenesis (Crespo et al.,2016). The pathway pI3k/AKT is one of the upstreams that regulate extracellular matrix metalloprotease (MMP2, MMP9) (Zhang et al.,2015). Consequently, the results of western blot showed that mocetinostat repressed the level of MMP2, MMP9 which are considered the enzymes involved in the degradation of the extracellular matrix(ECM) in glioblastoma and highly expressed in glioblastoma (Forsyth et al.,1999). Another study observed that expression MMP2 and MMP9 are controlled by expression Class I HDAC through deacetylating of transcription factor attaching to promoter of MMP2and MMP9. Hence, treatment of cells with HDACi caused decease the level expression of MMP2,MMP9 (Mani etai.,2015)

We also examined whether mocetinostat would suppress angiogenesis in glioblastoma cells or no, which is also one of the significant biological activities in the progression of glioblastoma cancer cells (Onishi et al.,2013). The angiogenesis in glioma cells is induced by increasing expression of pI3k/Akt protein and VEGF in glioma cells (Cea et al.,2012). The result of coculture HME- glioblastoma cell line C6 and T98G has been found that mocetinostat inhibited growth angiogenesis by inhibiting EGFR singling pathway promoted VEGF gene expression via HIF independent mechanism (Zhang et al.,2008). Another study also found that mocetinostat inhibits tubule growth of cultured human endothelial cells by inducing transcription of anti-angiogenesis factor, Thrombospondin-1 human cancer cell (Liu et al.,2008).

## Conclusion

In conclusion, our result in this study demonstrated the cytotoxic effects of different concentrations of mocetinostat (MGCD0103) on two glioblastoma cell lines C6 and T98G, and our finding in this study helped in understanding the molecular mechanism of MGCD0103 in induction the apoptosis and the differentiation in a manner dependent on dose and time. Moreover, the MGCD0103 played a vital role in suppressing migration, invasion, and angiogenesis on glioma. MGCD0103, a small molecular inhibitor, exerts anti-cancer activities against glioblastoma and is worth employing as a successful therapy to treat glioblastoma.

## Abbreviations

HDAC: Histon deacetylase
PI3K: Phosphatidylinositol-3-kinase
GFAP: Glial fibrillary acidic protein
Id2: Inhibitor of DNA Binding 2
DMSO: dimethyl sulfoxide
RPMI/1640 medium: Roswell Park Memorial Institute medium
HEPES: 4-(2-hydroxyethyl)-1-piperazineethanesulfonic acid
HME: Human Mammary Epithelial
BCA: Bicinchoninic acid
PVDF: Polyvinylidene fluoride
PBS: Phosphate-buffered saline
MMP-9: Matrix metalloproteinase −9
MMP-2: Matrix metalloproteinase −2
VEGF: Vascular endothelial growth factor
EGFR: Epidermal growth factor receptor
NF-κB: Nuclear factor kappa B
mTOR: Mammalian target of rapamycin
E2f6: E2 transcription factor6
Bax: Bcl2-associated X protein
Bcl-2: B-cell lymphoma 2 protein
TWIST1: Twist-related protein 1
TSA: Trichostatin
VPA: Valproic acid
HMVC: Human mammalian epithelia cell
PARP-1: Poly(ADP-ribose) polymerase-1
ICAD: A caspase-activated deoxyribonuclease inhibitor
ECM: Exocellular matrix
